# Inhibition of lipopolysaccharide (LPS)-induced neuroinflammatory response by polysaccharide fractions of *Khaya grandifoliola* (C.D.C.) stem bark, *Cryptolepis sanguinolenta* (Lindl.) Schltr and *Cymbopogon citratus* Stapf leaves in raw 264.7 macrophages and U87 glioblastoma cells

**DOI:** 10.1186/s12906-018-2156-2

**Published:** 2018-03-12

**Authors:** Francine Kengne Mediesse, Thaddée Boudjeko, Anantharaju Hasitha, Matharasala Gangadhar, Wilfred Fon Mbacham, Perumal Yogeeswari

**Affiliations:** 1 Laboratory of Phytoprotection and Valorisation of Plant Resources, The Biotechnology Centre, University of Yaounde I, P.O. Box 3851, Messa-Yaounde, Yaounde, Cameroon; 2 Drug Discovery Research Laboratory, Department of Pharmacy, Birla Institute of Technology and Science–Pilani, Hyderabad campus, Jawahar Nagar, Hyderabad, Andhra Pradesh –500078 India; 3 Department of Biochemistry, University of Yaounde I, P.O. Box 812, Yaounde, Cameroon; 4 Laboratory of Public Health Research Biotechnologies, The Biotechnology Centre, University of Yaounde I, P.O. Box 3851, Messa-Yaounde, Yaounde, Cameroon

**Keywords:** Plant polysaccharide, Khaya grandifoliola, Cryptolepis sanguinolenta, *Cymbopogon citratus*, Lipopolysaccharides, Anti-neuroinflammatory

## Abstract

**Background:**

*Khaya grandifoliola* (C.D.C.) stem bark, *Cymbopogon citratus* (Stapf) and *Cryptolepis sanguinolenta* (Lindl.) Schltr leaves are used in Cameroonian traditional medicine for the treatment of inflammatory diseases. Several studies have been performed on the biological activities of secondary metabolites extracted from these plants. However, to the best of our knowledge, the anti-neuro inflammatory and protective roles of the polysaccharides of these three plants have not yet been elucidated. This study aimed at investigating potential use of *K. grandifoliola*, *C. sanguinolenta* and *C. citratus* polysaccharides in the prevention of chronic inflammation.

**Methods:**

Firstly, the composition of polysaccharide fractions isolated from *K. grandifoliola* stem bark (KGF), *C. sanguinolenta* (CSF) and *C. citratus* (CCF) leaves was assessed. Secondly, the cytotoxicity was evaluated on Raw 264.7 macrophages and U87-MG glioblastoma cell lines by the MTT assay. This was followed by the in vitro evaluation of the ability of KGF, CSF and CCF to inhibit lipopolysaccharides (LPS) induced overproduction of various pro-inflammatory mediators (NO, ROS and IL1β, TNFα, IL6, NF-kB cytokines). This was done in Raw 264.7 and U87-MG cells. Finally, the in vitro protective effect of KGF, CSF and CCF against LPS-induced toxicity in the U87-MG cells was evaluated.

**Results:**

CCF was shown to mostly contain sugar and no polyphenol while KGP and CSP contained very few amounts of these metabolites (≤ 2%). The three polysaccharide fractions were non-toxic up to 100 μg.mL^− 1^. All the polysaccharides at 10 μg/mL inhibited NO production, but only KGF and CCF at 12.5 μg/mL down-regulated LPS-induced ROS overproduction. Finally, 100 μg/mL LPS reduced 50% of U87 cell viability, and pre-treatment with the three polysaccharides significantly increased the proliferation.

**Conclusion:**

These results suggest that the polysaccharides of *K. grandifoliola*, *C. citratus* and *C. sanguinolenta* could be beneficial in preventing/treating neurodegenerative diseases in which neuroinflammation is part of the pathophysiology.

## Background

Lipopolysaccharides (LPS) derived from gram-negative bacteria are considered to be the most potent activators of the production of various inflammatory mediators such as pro and anti-inflammatory cytokines, nitric oxide (NO) by macrophages [1–3]. LPS excreted during bacterial infection cause chronic neuroinflammation and progressive neurodegeneration [4, 5], therefore their stimulation is a good model for mimicking neuroinflammatory conditions [6]. Abrogation of the inflammatory mediator’s production may be the potential target(s) for neuroinflammatory therapeutics.

Children under the age of five affected by malaria are at risk of developing complications including severe anaemia and cerebral malaria (CM). CM occurs in non-immunized individuals and those with failure of standard antimalarial treatment [7]. It is mostly characterized by hyper-parasitemia, excessive production of pro-inflammatory cytokines (TNFα, INFγ, IL1β, IL6, IL12 etc.….) followed by up-regulation of endothelial cell adhesion molecule expression, which contribute to the sequestration of parasitized erythrocytes in the brain microvasculature [8]. This sequestration reduces the microvascular flow, thereby causing disruption of blood brain barrier (BBB), cerebral oedema and tissue hypoxia. Increasing evidence suggests that oxidative damage to cell components has a relevant pathophysiological role in several types of human diseases, including malaria. The inflammation of the brain may also contribute to a wide variety of neurodegenerative pathologies such as Alzheimer’s and Parkinson’s diseases, Amyotrophic Lateral Sclerosis, Multiples Sclerosis and psychiatric diseases [9, 10]. A lot of anti-inflammatory drugs are commercialised but due to their potential secondary effects (teratogenic, mutagenic, cancerigenic, gastric, metabolic, endocrinal, neuronal disorders), it is necessary to discover and develop much safer and new bioactive compounds [11].

According to Xie et al. [12], there is an increasing interest of pharmaceutical sectors and researchers in polysaccharides isolated from medicinal plants because of their biological activities that are antioxidant (inhibition of lipid peroxidation, free radicals scavenging activities, protection of DNA from breaks induced by ROS), anti-inflammatory, anticancer [13–15], as well as the stimulation of PBMC proliferation and INFγ cytokine production [16, 17]. Moreover, most polysaccharides derived from higher plants are relatively non-toxic and do not cause significant side effects as compared to immunomodulatory bacterial polysaccharides and synthetic compounds [18].

*Khaya grandifoliola* (Meliaceae family), *Cryptolepis sanguinolenta* (Periplocacees family) and *Cymbopogon citratus* (*Poaceae* family) are three medicinal plants found in Cameroon and many African countries. Decoctions from *K. grandifoliola* stem bark and root, *C. sanguinolenta* leaves and of *C. citratus* leaves are used in the treatment of malaria and other infectious diseases that provoke fever, pain and inflammation [19–21]. Numerous studies have been conducted in order to justify the folkloric use of these plants in the treatment of inflammatory diseases [11, 21–23]. The present work aimed at exploring the anti-neuroinflammatory (down-regulation of NO, ROS and various pro-inflammatory cytokines) of polysaccharide fractions isolated from these plants of interest. In vitro system experiment was performed using various spectrophotometry, cell biology and molecular biology techniques including: MTT assay, Griess reactions and DCFH-DA assay, and Quantitative Real Time PCR.

## Methods

### Materials

The stem barks of *Khaya grandifoliola* C.D.C. (Welw) (Meliaceae family), leaves of *Cymbopogon citratus* Stapf (Poaceae family) and *Cryptolepis sanguinolenta* (Lindl.) Schltr (Periplocaceae family) were collected from Mbalmayo forest, Emana and Ongot respectively, in the Centre Region of Cameroon. The plant species were identified and authenticated by Mr. Nana of the National Herbarium of Cameroon, Yaounde (Cameroon), (Voucher specimen no: 52658/SFR-Cam, N°14,243/HNC and 28,247/SRF-Cam respectively for *K. grandifoliola* stem bark, *C. citratus* and *C. sanguinolenta* leaves).

### Polysaccharide fraction preparation

The stem bark of *K. grandifoliola*, leaves of *C. citratus* and *C. sanguinolenta* were collected, shade-dried for one week at room temperature and powdered. Extraction of low molecular weight (LMW) polysaccharide fractions was performed as described by Thangam et al. [23] with slight modifications. Briefly, for the de-coloration and de-fatting process, 200 g of each powder was soaked in 60% methanol for 48 h in a shaker at 200 rpm. The resulting materials were boiled twice in 1 L of distilled water at 80 °C for 2 h and the supernatants were pooled and precipitated overnight at 4 °C with 95° ethanol (1:2 (*v*/v)). The polysaccharides were collected by centrifugation, then dissolved in water and dialyzed against distilled water at room temperature. Each dialysate was then mixed with 4:1 (v/v) CH_2_Cl_2_/ButOH solvent system and stirred for 15 min. After centrifugation, the upper polysaccharide solution was collected and deproteinized twice with CH_2_Cl_2_/ButOH solvent until there were no proteins left. The absence of proteins in each fraction was confirmed by the Bradford (1976) [24] method. The deproteinized polysaccharide solutions were then subjected to lyophilization and LMW polysaccharide fractions were obtained and were named KGF, CSF and CCF for *K. grandifoliola*, *C. sanguinolenta* and *C. citratus* respectively.

### Quantification of total sugar and phenolic compounds

Total sugars were determined using phenol-H_2_SO_4_ as described by Dubois et al. [25], where neutral monosaccharides were heated in acid medium and transformed into dehydrated derivatives of furfural. Practically, 0.2 mL of sample were mixed with 0.2 mL of 5% phenol. Then, 1 mL of concentrated sulfuric acid was added quickly and stirred. The mixture was placed at 100 °C for 10 min until it developed a yellow colour. The absorbance was read at 485 nm. The amount and level of sugar present were calculated using glucose as standard and expressed as μg equivalent of glucose (GE) per mg of dry polysaccharides.

Phenolic compounds were estimated by the Folin-Ciocalteu method [15]. Briefly, 750 μL of extract solution (0.3 mg/mL) of polysaccharide fractions were added to 75 μL of Folin-Ciocalteu reagent. After 3 min, 750 μL of Na_2_CO_3_ (20%) were added. The absorbance was measured at 760 nm using a UV-VIS 1605 Shimadzu spectrophotometer after 30 min in the dark. Phenolic compound amounts were calculated using ferulic acid as standard and expressed as μg equivalent of ferulic acid equivalent (FAE)/mg of dry polysaccharide.

### Toxicity of polysaccharide fractions on raw 264.7 macrophages and U87-MG cell lines

RAW 264.7 cells, the murine macrophage cell line and the U87-MG human glioblastoma cell line were obtained from the National Centre for Cell Science (NCCS) Pune, India. RAW 264.7 cells and U87-MG cells were cultured respectively in RPMI 1640 medium and Minimum Essential Medium (MEM) supplemented with 10% foetal bovine serum (FBS), L-Glutamine (200 mM), streptomycin (200 μg/ml) and penicillin (200 U/ml) and maintained at 37 °C in an atmosphere of 5% CO_2_. The assay was carried out on cells with 70% confluence. The cell viability was tested using Trypan Blue dye and at least 95% of viability was confirmed to carry out the assay.

RAW 264.7 cells (1 × 10^5^) were treated with 10 μg/mL LPS with or without different concentrations of polysaccharide fractions (100–1000 μg/mL) for 24 h (polysaccharide samples were added 1 h before LPS). Non-treated cells were utilized as negative controls and cells treated with LPS alone were utilized as positive controls. Aspirin, a non-steroid anti-inflammatory compound (previously shown to be non-toxic for Raw 264.7 cells), was used 1 μM as positive control. After treatment, the cell culture supernatants were collected for nitrite assay and plated cells used for cell proliferation assessment by MTT assay.

Otherwise, U87-MG (5 × 10^3^) cells were treated with different concentrations of polysaccharide fractions (100–1000 μg/mL) for 48 h and the cell proliferation was evaluated by MTT assay.

### Cell proliferation assay

Proliferation of cells after treatment was determined by the 3-(4, 5-dimethylthiazol-2-yl)-2, 5-Diphenyltetrazolium Bromide (MTT) colorimetric assay [26]. After incubation, the medium was discarded, MTT (0.5 mg/mL in PBS 1X) was added to the treated cells and the plate was incubated for an additional 4 h. The medium was discarded once more and Formazan Blue, which was formed in the cells, was dissolved with 100 μL of Dimethylsulphoxide (DMSO). The optical density (OD) was measured at 590 nm against a background at 620 nm using a microplate reader (Molecular Device Spectra M4, USA). The cell viability was determined as (Mean A1 _(590-620nm)_/Mean A0 _(590-620nm)_)*100 where A0 and A1 are respectively the absorbance in untreated and treated wells. The toxicity or growth inhibitory percentage of polysaccharide fractions were determined by the formula: 1-[(Mean A1 _(590-620nm)_ /Mean A0 _(590-620nm)_)]*100.

### In vitro evaluation of inhibitory effect of LPS-induced brain toxicity

An U87-MG glioblastoma cell line was used in this assay to mimic in vitro brain toxicity. For optimization of LPS dose, U87 cells were treated with LPS (1-200 μg/mL) and growth inhibitory percentage determined by MTT assay as previously described. The inhibitory concentration 50 (IC_50_) value was calculated using GraphPad Prism 6.01. The protective effect of polysaccharide fractions at non-toxic concentrations (12.5–100 μg/mL) on U87-MG was assessed by their co-incubation with LPS (100 μg/mL) for 48 h (polysaccharide samples were added 1 h before LPS). Diclofenac potassium at 50 μg/mL (previously shown to be non-toxic for U87-MG cells) was used as positive control. The medium was changed after 48 h and 100 μL of MTT (0.5 mg/ml stock solution) were added and the plates incubated for an additional 4 h. The medium was once more discarded and the Formazan Blue insoluble crystal, which was formed in the cells, was dissolved with 100 μL of DMSO. The OD was measured at 590 nm against a background at 620 nm. The protective effect of KGF, CSF and CCF was evaluated using GraphPad Prism 6.01.

### Nitrite assay

After treatment of RAW 264.7 cells with/without non-toxic concentrations of polysaccharide fractions and LPS, cell culture supernatant was used to quantify the NO secretion by the measurement of nitrite concentration in the supernatants using the Griess reaction [27]. Each culture supernatant was mixed with the same volume of Griess reagent 1X and the absorbance of the mixture at 540 nm was determined with a microplate reader (Molecular Device Spectra M4, USA). Fresh RPMI culture medium was used as the blank and the amount of nitrite in the samples was measured with the sodium nitrite serial dilution standard curve.

### Intracellular reactive oxygen species (iROS) estimation

Intracellular ROS estimation was measured using DCFH-DA [28]. Approximately 5 × 10^3^ U87-MG cells/well were plated on 96-well plates and treated with various polysaccharide fractions and/or LPS 10 μg/mL for 24 h. Then, the plates were washed with PBS 1X cells were treated with 10 μM DCFH-DA for 1 h. The loading buffer was removed and the plates washed. New PBS 1X was added into each well and oxidized DCFH excitation and emission were measured at 485 and 525 nm respectively in a spectrophotometer (SpectraMax M4, Molecular Devices, Sunnyvale, USA). Diclofenac potassium at non-toxic concentration on U87-MG was used as positive control. Percentage ROS production inhibition was computed using Graph Pad prism 6.01.

### In vitro evaluation of inhibitory effect of LPS-induced overexpression of pro-inflammatory cytokines

About 10^5^ U87-MG cells/well/2 mL in 96 well plates were treated with polysaccharide fractions for 1 h followed by 100 μg/mL LPS for 48 h. After incubation, cells were washed with 500 μL of PBS 1X, and total RNA was extracted from the untreated cells and from the LPS and/or polysaccharide fraction treated cells using RNA Xpress reagent according to the manufacturer’s protocol. RNA was then quantified with Nanodrop and purity of RNA was determined with A_260_/A_280_ ratio (1.8–2.0 was considered pure). RNA (1 μg) was reverse-transcribed using a cDNA synthesis kit. Quantitative Real Time PCR was performed on reversed-transcribed cDNA products for determination of TNF-α, IL6, IL-1β and NF-kB expression according to the manufacturer’s instructions using a BIO-RAD CFX Connect, with SYBR Green (Kapa Biosystems) as the fluorescent dye, enabling real-time detection of PCR products. Sense and antisense PCR primers used in this study were purchased from Sigma Genosis, India (Table 1). All samples were run in triplicates and the output values reported were the average of three wells. The amplification consisted of denaturation at 95 °C for 25 s, primer annealing at 58–64 °C for 30 s and extension at 72 °C for 1 min, for a total of 37 cycles followed by final extension at 72 °C for 10 min. For quantification, the target gene was normalized to the internal standard GAPDH gene.

**Table 1:** Details of primer sequences (Sigma Genosys) used for inflammatory mediators gene expression through RT-PCR amplification

Genes	Oligoname	Oligonucleotide sequence 5′…………… ………………..3′	Length	Anneal T°
IL6	Human-IL6-FP	TTCGGTCCAGTTGCCTTCTC	20	61.8 °C
	Human-IL6-RP	GAGGTGAGTGGCTGTCTGTG	20	
TNFα	Human-TNFalpha-FP	CTCCAGGCGGTGCCTTGTTC	20	60.4 °C
	H-TNFalpha-RP	CAGGCAGAAGAGCGTGGTG	19	
IL1β	Human-IL1beta-FP	GCAAGGGCTTCAGGCAGGCCGCG	23	64 °C
	Human-IL1beta-RP	GGTCATTCTCCTGGAAGGTCTGTGGGC	27	
NF-kB	Human-Nf-kB-FP	GCGCTTCTCTGCCTTCCTTA	20	58 °C
	Human-Nf-kB-RP	TCTTCAGGTTTGATGCCCCC	20	
GAPDH	Human-GAPDH-FP	ACCACAGTCCATGCCATCAC	20	60.4 °C
	Human-GAPDH-RP	TCCACCACCCTGTTGCTGT	19	

### Statistical analysis

Results were expressed as the means ± SEM. Multi group comparison was performed by one-way analysis of variance, followed by the Dunnet’s multiple comparisons as a post hoc analysis test for comparison between polysaccharide treatment and positive control, non-treated or LPS group at *p* < 0.05, *p* < 0.01 and *p* < 0.001. Calculations were performed using GraphPad InStat® version 6.01 software. IC_50_ was analyzed using non-linear regression.

## Results

### Partial composition and in vitro toxicity of polysaccharide fractions

After the extraction procedure that included deproteinization, carbohydrates were the major components in the polysaccharide fractions. *C. citratus* polysaccharide presents the highest level of sugar (629.78 ± 1.4 μg GE/mg of dry polysaccharide) and no polyphenols. However, small amount of polyphenols were found in KGF and CSF polysaccharides (less than 2%) (Table 2). Therefore, the potential of these polysaccharide fractions could be related only to their carbohydrate content.

**Table 2:** Total sugar and polyphenols content of polysaccharides fractions of *Khaya grandifoliola* stem bark (KGF), *Cryptolepis sanguinolenta* (CSF) and *Cymbopogon citratus* (CCF) leaves by Phenol-sulfuric and Folin-Ciocalteu methods (mean ± SEM; n = 3)

Polysaccharide fractions	Total sugar content (μg GE/mg)	Total polyphenol (μg FAE/mg)
KGF	450.09 ± 0.33	17.76 ± 0.15
CSF	437.19 ± 0.53	15.54 ± 0.5
CCF	629.78 ± 1.39	0

**Fig. 1:**
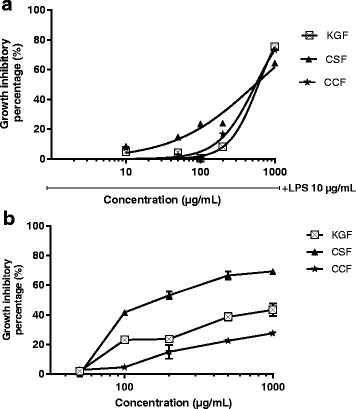
Cytotoxicity of polysaccharide fractions of *Khaya grandifoliola* stem bark (KGF), *Cryptolepis sanguinolenta* (CSF) and *Cymbopogon citratus* (CCF) leaves on RAW 264.7 macrophages cell lines (**a**) and U87-MG glioblastoma cell line (**b**)

In order to assess the effects of all the three polysaccharide fractions on the viability of RAW 264.7 and U87-MG cells, the cells were treated with the indicated concentrations of KGF, CSF and CCF in the presence or absence of LPS and cell viability analysed by MTT assay. The results were shown in Fig. 1. The viability of RAW 264.7 cells was not significantly altered after 24 h of incubation with up to 100 μg/ml of polysaccharide fractions (Fig. 1) and the data revealed noticeable toxicity on U87 MG for all the fractions tested with less than 100 μg/mL for 48 h (*p* < 0.001). Up to 100 μg/mL, all the polysaccharide fractions significantly altered the two cells viability in a dose-dependent manner and cell viability decreased with increasing concentration. A growth inhibitory concentration of 80% was observed at 1 mg/mL. The results of the IC_50_ concentrations for RAW 264.7 and U87-MG cells with respect to all the three fractions are shown in Table 3. Therefore, in subsequent experiments, the concentrations of polysaccharide fractions used were 10 to 100 μg/mL.

**Table 3:** Inhibitory concentration 50 for Raw 264.7 Macrophages and U87-MG Glioblastoma cells line with respect to polysaccharide fractions of *K. grandifoliola* stem bark*, C. sanguinolenta* and *C. citratus* leaves

Polysaccharide fractions	IC50 (μg/mL) for Raw 264.7 cells	IC50 (μg/mL) for U87-MG
KGF	600.9	›1000
CSF	545	243.7
CCF	554.1	›1000

### Effect of KGF, CSF and CCF in LPS induced anti-proliferation in the U87-MG cell lines

**Fig. 2:**
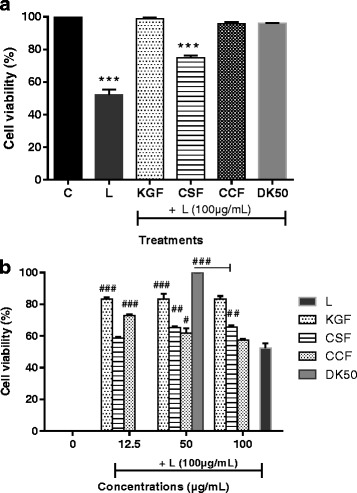
Inhibition of LPS toxicity on U87 cell lines by various polysaccharide fractions and diclofenac potassium; (**a**) C-control cells; L-LPS (100 μg/mL), KGF, CSF, CCF polysaccharides of *Khaya grandifoliola* stem bark, *Cryptolepis sanguinolenta* and *Cymbopogon citratus* leaves were tested at 100 μg/mL and Diclofenac potassium at 50 μg/mL (DK50). Data are expressed as mean ± SEM, *** denote statistical significance *p* < 0.001 in comparison to the control group; (**b**) KGF, CSF, CCF polysaccharides were tested at 12.5, 50 and 100 μg/mL and DK50 #, ##, ### denote statistical significance at *p* < 0.05, *p* < 0.01 & *p* < 0.001 in comparison to the LPS treated group

The protective effect of KGF, CSF and CCF over LPS induced toxicity was evaluated through MTT assay. The protective effect of polysaccharide fractions is their ability to inhibit LPS toxicity and increase U87-MG cell proliferation. The IC_50_ for LPS in the U87-MG was found to be 100 μg/mL. LPS (100 μg/mL) treatment significantly decreased cell viability from 100 to 52.3 ± 3.1% but polysaccharides co-treatment increased the U87-MG proliferation and viability (*p* < 0.001). According to the concentrations of the test fractions, KGF and CCF were most active at 12.5 μg/mL (cell viability up to 83.3 ± 0.2 and 72.9 ± 0.8% respectively) (Fig. 2). The protective effect of CSF was observed at 50 μg/mL (cell viability up to 65.21 ± 0.72%). However, no dose-dependent linear effect was observed. Compared to polysaccharides, diclofenac potassium at 50 μg/mL was non-toxic on U87-MG cells (4.8 ± 0.8% of growth inhibitory percentage) and presented the best protective effect (cell viability up to 99.97 ± 0.03%) (*p* < 0.001).

### Effect of KGF, CSF and CCF on NO and ROS secretion

**Fig. 3:**
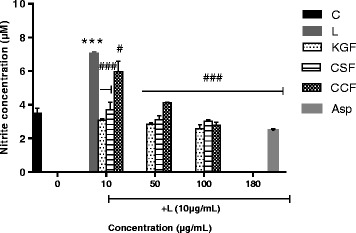
Effects of polysaccharides fractions of *Khaya grandifoliola* stem bark (KGF), *Cryptolepis sanguinolenta* (CSF) and *Cymbopogon citratus* (CCF) leaves and Aspirin (Asp) on the secretion of NO by LPS-stimulated RAW 264.7 macrophages cells. C-Non-treated cells; L-LPS (10 μg/mL) treated cells, Asp-Aspirin 1 μM (180 μg/mL) treated cells, Data expressed as mean ± SEM (*n* = 3). *, **, *** denote statistical significance at *p* < 0.05, *p* < 0.01 & *p* < 0.001 in comparison to the control group. #, ##, ### denote statistical significance at *p *< 0.05, *p* < 0.01 & *p* < 0.001 in comparison to the LPS treated group (Dunnet multiple comparison, graph Pad Prism 6.0)

**Fig. 4:**
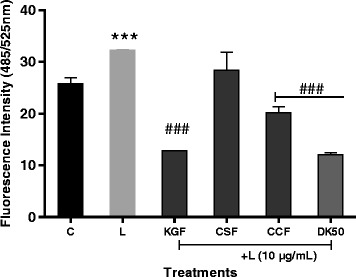
Effects of polysaccharides isolate from *Khaya grandifoliola* stem bark (KGF), *Cryptolepis sanguinolenta* (CSF) and *Cymbopogon citratus* (CCF) leaves at 12.5 μg/mL and Diclofenac potassium on the secretion of ROS by LPS-stimulated U87-MG cells. C-control cells; L-LPS (10 μg/ml), DK50-Diclofenac potassium 50 μg/mL. Data expressed as mean ± SEM (n = 3). *, **, *** denote statistical significance at *p* < 0.05, *p* < 0.01 & *p* < 0.001 in comparison to the control group. #, ##, ### denote statistical significance at *p* < 0.05, *p* < 0.01 & *p *< 0.001 in comparison to the LPS treated group (Dunnet multiple comparaison, graph Pad Prism 6.0)

To investigate whether polysaccharide fractions regulate NO production, RAW 264.7 cells were pretreated with KGF, CSF and CCF for 1 h before treatment with LPS for 24 h, and nitrite content, a stable end product of NO was measured using Griess reaction. Treatment with LPS resulted in significant up-regulation of nitrite production (7.05 ± 0.09 μM), compared to the untreated control (3.5 ± 0.3 μM) (Fig. 3). However, RAW 264.7 cells pretreated with all the polysaccharide fractions at 100 μg/mL displayed a marked decrease in the induction of nitrite after stimulation with LPS (*p* < 0.001). The nitrite production were respectively 2.56 ± 0.25; 3.02 ± 0.09 and 2.8 ± 0.16 μM for KGF, CSF and CCF treated cells at 100 μg/mL. The growth inhibitory percentage of Aspirin at 1 μM (180 μg/mL) on Raw 264.7 cells was 0.98 ± 0.28%. At this concentration, Aspirin-treated cells produced 2.46 ± 0.1 μM of nitrite. On the other hand, estimation of intracellular ROS was performed using DCFH-DA. The U87-MG cell treated with 10 μg/mL of LPS showed increased DCF fluorescence levels (compared to the control, *P <* 0.005) indicating of intracellular ROS production (Fig. 4). However, pre-incubation with KGF and CCF polysaccharide (12.5 μg/mL) and Diclofenac potassium (50 μg/mL) for 1 h before LPS treatment decreased the fluorescence intensity of the cells. CSF polysaccharide did not reduce intracellular ROS in LPS-induced U87-MG. The most promising KGF and CSF showed the best NO and ROS inhibitory effects in LPS stimulated RAW 264.7 and U87-MG cell lines.

### Effect of polysaccharide fractions on mRNA expression of IL-1β, IL6, NF-kB and TNFα

**Fig. 5:**
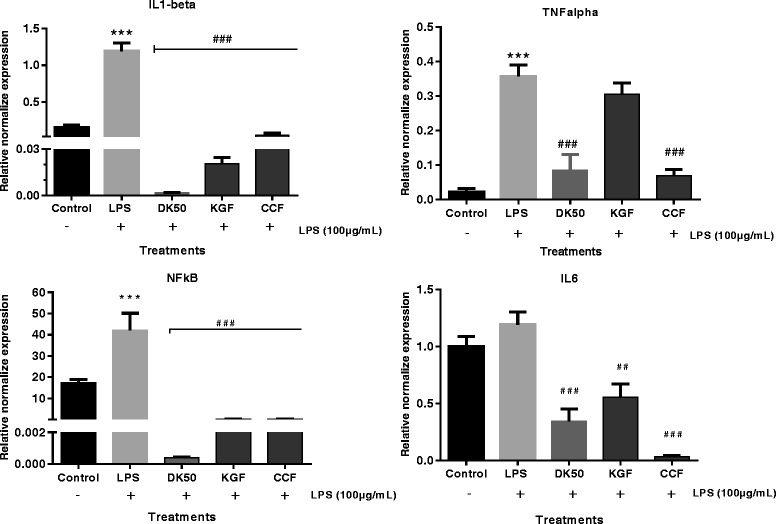
Normalised gene expression levels of proinflammatory cytokines in LPS induced U87-MG cell lines (n = 3). The mRNA expression values were given as mean ± SEM normalised to GAPDH levels in each sample. Y-axis values represent the number of mRNA copies relative to the number of GAPDH copies. Control-untreated cell line; LPS (100 μg/mL), *K. grandifoliola* stem bark (FKG) and *C. citratus* leaves (FCC) were tested at 12.5 μg/mL, DK50-Diclofenac potassium 50 μg/mL treated cells. Data are expressed as mean ± SEM. #, ##, ### denote statistical significance at *p* < 0.05, *p* < 0.01 & *p* < 0.001 in comparison to the LPS treated group. *, **, *** denote statistical significance at *p* < 0.05, *p* < 0.01 & *p* < 0.001 in comparison to the control group

The systematic study of simultaneous changes in gene expression for several pro-inflammatory cytokines in LPS-induced U87-MG cell lines was performed using Real Time gene expression studies. Comparing the LPS-treated and untreated cells, IL-1β, NF-kB, and TNF-α expression showed significant up-regulation. KGF and CCF polysaccharides at 12.5 μg/mL were able to significantly reduce the over-expressions of IL-6, IL-1β, NF-kB but only CCF reduced TNF-α over-expression. KGF and CCF suppressed the production of IL-6 and IL-1β in LPS-induced U87-MG cell lines by inhibiting NF-κB activation which is an important signaling pathway involved in the production of cytokines. Diclofenac potassium at 50 μg/mL was more active on IL-1β and NF-kB compared to the polysaccharides (Fig. 5).

## Discussion

Polysaccharides are present in plants, animals and microorganisms bound to with proteins and phenolic compounds. During their extraction, these others compounds are also obtained in various concentrations. Phenolic compounds are known to have several biological activities including antioxidant and anti-inflammatory properties [29]. According to Chen et al. [30] and Mediesse et al. [15], the bioactivity of polysaccharide extracts increases in a dose-dependent manner with protein, phenolic and lipid contents. Recent studies now show that pure polysaccharides also have several biological activities [23, 31]. Some research has been oriented towards the identification of pure and bioactive polysaccharides. In this study, the modulation of *K. grandifoliola, C. sanguinolenta and C. citratus* polysaccharide fractions for LPS-induced inflammatory responses were examined in vitro. From cytotoxicity experiments, it was observed that the CSF polysaccharide exhibited more significant cytotoxic effect against the cancer cells than KGF and CCF fractions. The concentrations of KGF, CSF and CCF polysaccharides (≤ 100 μg/mL) used in this study were considered to be non-cytotoxic.

During inflammation, macrophages induce the expression of pro-inflammatory genes such as inducible nitric oxide synthase (iNOS). Cytokines, such as interleukin (IL)-1, IL-6, and tumor necrosis factor (TNF), have been known to play important roles in pro-inflammatory response [32, 33]; and iNOS is up-regulated by secretion of pro inflammatory cytokines and produces nitric oxide from L-arginine. Because high concentrations of NO can be toxic, the regulation of NO production is therefore an important target for inflammatory disease [34]. Our results indicate that FKG, FCS and FCC treatment can reduce NO in the RAW 264.7 cell. Pacheco-sanchez et al. [35] also reported that polysaccharides extracted from *Collybia dryophila* treatment showed a down-regulation effect of NO production. According to Kim et al. [36] classification, the percentage of NO inhibition from plant extract represents its anti-inflammatory potential. Therefore KGF and CCF polysaccharides could be considered as moderate and CSF polysaccharide as weak anti-inflammatory agent. In addition, LPS stimulates various transcription factors especially NF-κB which causes a chain of mechanisms, resulting in the production of pro-inflammatory cytokines TNFα, IL1β, and IL6 and inflammatory mediator iNOS [37, 38]. Persistent NF-κB activation also causes chronic inflammation, which has long been related to certain types of cancers [39, 40]. In this study, after LPS incubation with U87 glioblastoma cell, the pro-inflammatory cytokine TNFα, IL1β, and IL6 levels were elevated; the response was reversed by co-incubation of polysaccharide fractions in LPS. Several studies suggested that the biological activities of polysaccharides would strongly depend on their monosaccharide composition, structure, the degree of sulfation, the molecular weight, the sulfation pattern, and the glycosidic branches [41]. Luhm et al. [42] reported that β (1 → 3) -D-Glucans from plant material were shown to be capable of having beneficial effects in pre-inflammatory responses, indicating that β-glucan can be a modulator of the anti-inflammatory response as interleukin mediators. We do not exactly understand the mechanism by which the KGF, CSF and CCF treatment reduce inflammation in the RAW 264.7 and U87 cells. Therefore, more research is needed to understand the effects of treatment with *K. grandifoliola, C. sanguinolenta* and *C. citratus* polysaccharide fractions on anti-inflammation and how these three polysaccharide fractions relate to changes of cytokine, ROS and NO.

## Conclusion

The present study was conducted to explore the potential use of *K. grandifoliola*, *C. sanguinolenta* and *C. citratus* polysaccharides to prevent chronic neuroinflammation. The results of the study show that KGF, CSF and CCF inhibit the toxic effect of LPS, increase brain cell (U87-MG) proliferation and exert anti-inflammatory effect by regulating various pro-inflammatory mediators (NO, ROS and pro-inflammatory cytokines). However, further detailed experiments need to be performed to understand the exact mechanisms by which they act. Thus, KGF and CCF particularly present the best activities which could be used in a variety of painful conditions and also to attenuate neuroinflammation in neurological disorders.

## Abbreviations

CNS: Central Nervous System
DCFH-DA: Dichlorofluorescein diacetate
FCC: Polysaccharide fraction of *Cymbopogon citratus*
FCS: Polysaccharide fraction of *Cryptolepis sanguinolenta*
FKG: Polysaccharide fraction of *Khaya grandifoliola*
GAPDH: Glyceraldehyde phosphate dehydrogenase
INFγ: Interferon gamma
iROS: intracellular reactive oxygen
LPS: Lipopolysaccharide
MEM: Minimum Essential Medium
MTT: 3-(4,5-dimethylthiazol-2-yl)-2,5-diphenyltetrazolium bromide
NF-kB: Nuclear factor kappa B
NO: Nitrite oxide
TNFα: Tumor Necrosis Factor α
